# An isolated beating pig heart platform for a comprehensive evaluation of intracardiac blood flow with 4D flow MRI: a feasibility study

**DOI:** 10.1186/s41747-019-0114-5

**Published:** 2019-10-25

**Authors:** Eva S. Peper, Alberto M. Leopaldi, Sjoerd van Tuijl, Bram F. Coolen, Gustav J. Strijkers, Jan Baan, R. Nils Planken, Arend de Weger, Aart J. Nederveen, Henk A. Marquering, Pim van Ooij

**Affiliations:** 1 Department of Radiology and Nuclear Medicine, Amsterdam UMC, University of Amsterdam, Amsterdam, The Netherlands; 2 LifeTec Group, Eindhoven, The Netherlands; 3 Department of Biomedical Engineering & Physics, Amsterdam UMC, University of Amsterdam, Amsterdam, The Netherlands; 4 Department of Cardiology, Amsterdam UMC, University of Amsterdam, Amsterdam, The Netherlands; 5 Department of Cardiothoracic Surgery, Leiden University Medical Center, Leiden, The Netherlands

**Keywords:** Heart valve diseases, Isolated heart preparation, Magnetic resonance imaging (4D flow), Swine, Transcatheter aortic valve replacement

## Abstract

**Background:**

Cardiac magnetic resonance imaging (MRI) in large animals is cumbersome for various reasons, including ethical considerations, costs of housing and maintenance, and need for anaesthesia. Our primary purpose was to show the feasibility of an isolated beating pig heart model for four-dimensional (4D) flow MRI for investigating intracardiac blood flow patterns and flow parameters using slaughterhouse side products. In addition, the feasibility of evaluating transcatheter aortic valve replacement (TAVR) in the model was investigated.

**Methods:**

Seven slaughterhouse pig hearts were installed in the MRI-compatible isolated beating pig heart platform. First, Langendorff perfusion mode was established; then, the system switched to working mode, in which blood was actively pumped by the left ventricle. A pacemaker ensured a stable HR during 3-T MRI scanning. All hearts were submitted to human physiological conditions of cardiac output and stayed vital for several hours. Aortic flow was measured from which stroke volume, cardiac output, and regurgitation fraction were calculated.

**Results:**

4D flow MRI acquisitions were successfully conducted in all hearts. Stroke volume was 31 ± 6 mL (mean ± standard deviation), cardiac output 3.3 ± 0.9 L/min, and regurgitation fraction 16% ± 9%. With 4D flow, intracardiac and coronary flow patterns could be visualised in all hearts. In addition, we could study valve function and regurgitation in two hearts after TAVR.

**Conclusions:**

The feasibility of 4D flow MRI in an isolated beating pig heart loaded to physiological conditions was demonstrated. The platform is promising for preclinical assessment of cardiac blood flow and function.

**Electronic supplementary material:**

The online version of this article (10.1186/s41747-019-0114-5) contains supplementary material, which is available to authorized users.

## Key points

- An isolated beating pig heart MRI-compatible model was prepared and submitted to human physiological conditions of cardiac output.
- In seven pig hearts, four-dimensional flow sequences were successfully acquired and intracardiac and coronary flow patterns could be visualised.
- In two hearts, valve function and regurgitation after TAVR were also studied.

## Background

Large animal models, in particular sheep and pigs, have provided indispensable and valuable insights in cardiac anatomy and physiology during health and disease, as they are easy to manipulate and to reproduce [1]. Additionally, working animal models have demonstrated to be realistic training models for cardiologic intervention [2]. However, laboratory animal husbandry of sheep or pigs is expensive, and medical experiments with large animals are subject to increased ethical objections in many countries. The use of isolated pig hearts harvested from pigs slaughtered for human consumption might be an acceptable alternative, as it leads to a reduction in the use of live animals. Additionally, isolated hearts have the advantage of controlled physiological conditions (*e.g*., controlled blood pressure) and experimental settings (*e.g*., surgical intervention or drug administration) [3].

A promising non-invasive imaging modality to measure three-dimensional (3D) blood flow is the time-resolved three-dimensional phase-contrast magnetic resonance imaging (MRI), *i.e*., four-dimensional (4D) flow MRI [4, 5]. This technique facilitates accurate 3D visualisation and quantification of blood flow, in conjunction with the quantification of cardiac parameters such as cardiac output (CO), stroke volume (SV), and regurgitation fraction [6]. Studies showed that 4D flow MRI is valuable for intracardiac blood flow visualisation [7], vortex detection [8], and flow assessment through all four heart valves [9, 10], as well as for detecting flow alterations after TAVR and other surgical procedures in the aorta [11–14]. Traditionally, transthoracic or transesophageal echocardiography is used to follow-up after surgical interventions like transcatheter aortic valve replacement (TAVR) [15]. However, it remains challenging to measure the extent and type of regurgitation or deviations in flow patterns with echocardiography [15, 16].

Using 4D flow MRI in animal models, *in vivo* experiments of juvenile pigs have demonstrated that intraventricular flow patterns change under drug-induced stress [17]. This technique has also revealed disturbed left ventricle (LV) flow patterns after mitral annuloplasty in sheep, correlated with the size of the annuloplasty ring [18].

In this study, we introduce an MRI-compatible platform for 4D flow measurements in isolated beating pig hearts, with real blood actively pumped by the LV at physiological pressures. This provides *in vivo* blood flow behaviour, resulting in physiological coronary flow and myocardial perfusion with good-to-excellent MRI contrast [3, 19]. In a previous study, Schuster et al. [3] presented an MRI-compatible isolated beating pig heart model to investigate cardiac perfusion. Its design was based on the Langendorff model [20], which allows for coronary perfusion and LV contraction, but not for LV filling and output. The working heart model presented in this study is similar to that described by Vaillant et al. [21]. It actively pumps blood in both ventricles, which, in contrast to Langendorff perfusion, causes natural coronary filling, mimicking physiological heart function and providing real control on the relevant physiological parameters.

We investigate the feasibility and reproducibility of 4D flow MRI in an *ex vivo* beating pig heart platform by measuring flow parameters and performing flow visualisation in five repeated pig heart experiments with native valves. In two additional experiments, we test the feasibility of the working platform for evaluating transcatheter aortic valve replacement (TAVR) procedures. We hypothesised that this platform may help investigate the influence of different surgical procedures on intracardiac flow and the performance of MRI sequences in different pathophysiological settings, without needing a live animal experiment.

## Methods

### Preparation

In this study, seven hearts were retrieved from pigs slaughtered for human consumption (Dutch Landrace hybrids, approximately 110 kg live weight). The protocols at the slaughterhouse and during the experiment were in agreement with European Community regulations 1069/2009 and 142/2011 regarding the use of slaughterhouse byproducts for research and were approved by the associated legal authorities of animal welfare. 4D flow MRI was applied in five pig hearts with native valves. In two additional experiments described in Additional file 3, the native values of two other hearts were replaced in the preparation phase of the experiment with TAVR valves by a specialist (A.d.W.) with 12 years of experience. All experimental and post-processing steps were the same as for hearts with native values. The prosthetic valves available were the CoreValve (29 mm, Medtronic Inc., Minneapolis, MN, USA) and Edwards SAPIENS XT (26 mm, Edwards Lifesciences LLC, Irvine, CA, USA). The prosthetic valve sizes were not selected according to the pigs’ annulus size, which is why the Edwards SAPIENS XT valve was additionally stabilised by a purse string suture.

Each slaughterhouse pig heart was harvested and arrested with crystalloid cardioplegic solution after a very short warm ischaemic time. Approximately 10 L of blood was collected from the same animal and heparinised*.* The heart was transported in an iced cardioplegic solution and connected to the MRI-compatible isolated beating pig heart platform [19] (PhysioHeart^TM^ platform, LifeTec Group BV, Eindhoven, The Netherlands) on average 4 h after death. During preparation, the pericardial sac was discarded and the right pulmonary veins, vena cava inferior, vena cava superior, and the left azygos vein were tied off. A 27-mm cannula was inserted into the opened left pulmonary vein and secured with a purse string suture. The connecting tubes were compliant for blood flow and connected to preload simulating pulmonary resistances. A cannula with an internal diameter of 24 mm was inserted into the ascending aorta and fixed downstream to the aortic valve.

**Fig. 1:**
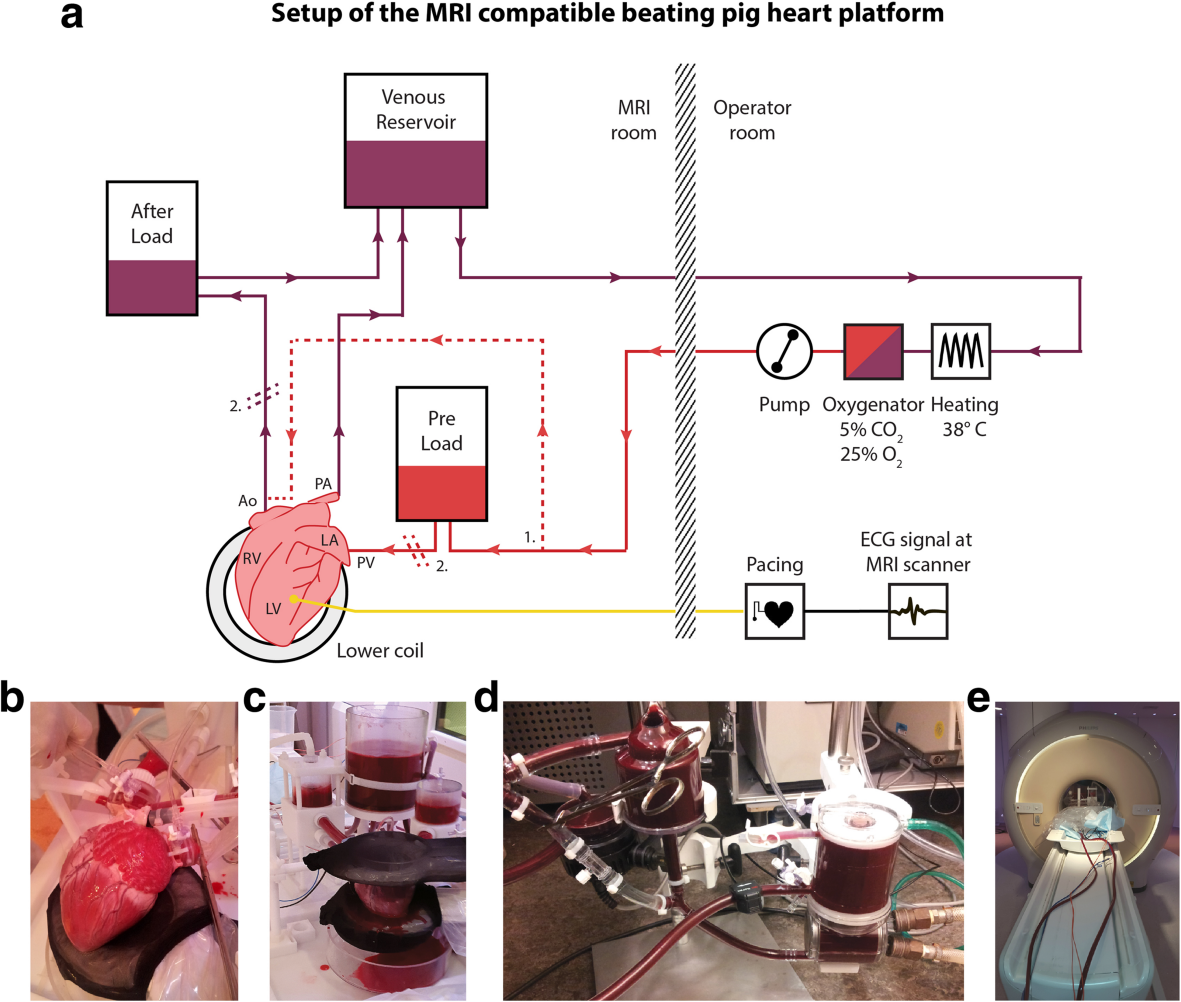
Experimental setup of the MRI-compatible beating pig heart platform. **a** Experimental setup of the MRI-compatible PhysioHeart^TM^ platform (LifeTec, Eindhoven, The Netherlands). The heart was connected to a preload and afterload system. The venous blood (purple) was heated and oxygenated outside the MRI room and pumped back to the experiment afterwards (red). The dotted lines indicate (1) coronary perfusion during the Langendorff mode and (2) the cross clamping of the aorta and the pulmonary vein. *Ao* Aorta, *LA* Left atrium, *LV* Left ventricle, *PA* Pulmonary artery, *PV* Pulmonary vein, *RV* Right ventricle. **b** The heart during Langendorff perfusion, attached to the aortic cannula and the pulmonary vein cannula. **c** The resuscitated, beating pig heart installed in the platform between the two coils on the patient bed before scanning. **d** The heating device and oxygenator outside the MRI room. **e** Setup during the scan, with two tubes leading to the MRI operator room

The aortic tube drained in an afterload simulating body resistance (systolic/diastolic pressure approximately 120/80 mmHg). A CO of approximately 4.5 L/min was maintained during the experiments to match human conditions. The CO was real time controlled via a flow sensor and regulated by adapting the preload and afterload resistances. A 17-mm cannula was inserted in the pulmonary artery, which was connected directly to a reservoir. The balanced blood was warmed and oxygenated by a heart lung machine outside the MRI room. The blood returned as a nutrient-rich, warm, oxygenated solution of 5% CO_2_ and 25% O_2_ and with a temperature of 38 °C. A schematic overview of the connections of pre- and afterload is shown in Fig. 1a.

### Reperfusion

To resuscitate the heart, a Langendorff perfusion [20] mode was created by cross clamping preload and afterload systems (Fig. 1b). Via a side port in the aortic cannula, blood was pumped retrograde in the aorta at approximately 80 mmHg closing off the aortic valve forcing flow into the coronary system. After perfusion of the myocardium, the deoxygenated blood drained in the coronary sinus, the right atrium, and the right ventricle. Through the pulmonary artery, blood was pumped back to the reservoir. The heart was left in Langendorff perfusion to recover for about 30 min until a steady state (in terms of a physiological colour and temperature, a stable sinus rhythm, a constant coronary flow, and constant pressures) was obtained (Additional file 1: Video S1).

Hereafter, the platform was switched to the working mode (Fig. 1c): Langendorff perfusion was stopped and the aorta and left pulmonary vein (preload) were opened, resulting in blood actively pumped by the LV with sufficient preload leading to a physiological CO. Coronary filling was created by LV function and aortic pressure only. To ensure a stable heart rate (HR) during MRI scanning, a pacemaker was attached to the heart. The paced HR was approximately 5–10 beats per min (bpm) over the irregular HR of the resuscitated heart. As soon as the heart was beating with a stable rhythm (approximately 20 min after switching from the Langendorff mode to the working mode), the setup was inserted into the MRI scanner (Fig. 1e) and the acquisitions were started.

### 4D flow MRI

All acquisitions were performed with a 3-T scanner (Ingenia, Philips Healthcare, Best, The Netherlands) using retrospectively triggered 4D flow MRI. Two medium flex coils (diameter 10 cm) were connected to the isolated beating pig heart platform below and above the heart (Fig. 1c). Electrocardiography sensors were attached via copper wires on the hearts’ surface and were used as a cardiac trigger signal. Twenty-four cardiac frames at a temporal resolution of 21 ms were acquired covering the cardiac cycle.

The 4D flow MRI scan had a field of view of 150 × 150 × 150 mm^3^ and a non-interpolated spatial resolution of 2.3 × 2.3 × 2.3 mm^3^. Echo time, repetition time, and flip angle were 2.2 ms, 5.2 ms, and 8°, respectively. To reduce the typically long acquisition times of 4D flow MRI, the scan was accelerated three times using k-t principal component analysis (Gyrotools, Zürich, Switzerland) resulting in a scan time of 12 min [22]. k-t principal component analysis acquisitions undersample k-space regularly over time, together with an interleaved training scan of the k-t space centre. The images are recovered during reconstruction by exploiting the relevant signal correlations available in the training data, which are represented as temporal basis functions and can be derived using a principal component analysis. The data was reconstructed using CRecon (Gyrotools, Zürich, Switzerland) with a k-t regularisation parameter of *λ* = 1. The velocity encoding was 100 cm/s.

### Flow measurements

Visualisation of 4D flow MRI data and quantification of velocity and flow were done with GTFlow (Gyrotools, Zurich, Switzerland). For flow calculation, a region of interest (ROI) was chosen in the ascending aorta close to the sinotubular junction and downstream to the aortic valve. Net flow, forward flow, and backward flow were determined. The net flow was defined as the spatially averaged flow through the defined ROI:

$$ Q(t)={\int}_{\mathrm{ROI}}v\left(\mathbf{r},t\right)\ {d}^2\mathbf{r} $$

with *v*(**r**, *t*) being the velocity (pointing either in forward or backward direction) at position **r** and cardiac phase *t*.

Likewise, forward was defined as:

$$ Q{(t)}_{\mathrm{forward}}={\int}_{\mathrm{ROI}}v{\left(\mathbf{r},t\right)}_{v>0}\ {d}^2\mathbf{r} $$

and backward flow was defined as:

$$ Q{(t)}_{\mathrm{backward}}={\int}_{\mathrm{ROI}}v{\left(\mathbf{r},t\right)}_{v<0}\ {d}^2\mathbf{r}. $$

For the per cent quantification of the regurgitation fraction for one cardiac cycle *T*, forward flow volume was calculated as

$$ {V}_{\mathrm{forward}}={\int}_0^TQ{(t)}_{\mathrm{forward}}\  dt $$

and backward flow volume was calculated as:

$$ {V}_{\mathrm{backward}}={\int}_0^TQ{(t)}_{\mathrm{backward}}\  dt. $$

The per cent regurgitation fraction was based on the ratio RF = *V*_backward_/|*V*_forward_|100. The stroke volume in millilitres was defined as SV_flow_ = *V*_forward_ − |*V*_backward_|. The CO_flow_ in litres per minute was calculated by the product of the mean HR during the acquisition and SV_flow_.

In one heart, the 4D flow MRI scan was repeated three times within the same scan session at time points 0 min, 12 min, and 1 h 5 min.

### Volume measurements

The LV volume was quantified for each heart using a 4D segmentation tool of velocity data from Medis (Medis medical imaging systems, Leiden, The Netherlands). Stroke volume was calculated as the difference between end-diastolic volume (EDV) and end-systolic volume (ESV). The per cent ejection fraction (EF) was defined as EF = SV_volume_ × 100/EDV. The size (long axis, short axis, and area) of the aortic annulus was measured during systole at the height of the aortic valve. Cardiac output was similarly calculated as the product of mean HR and SV_volume_.

### Statistical analysis

A statistical comparison between the flow and volumetric measurements was done using a Bland-Altman analysis and linear regression. Normal distribution of the data was tested using a Shapiro-Wilk test. The significance level was set to *p* < 0.05.

## Results

**Fig. 2:**
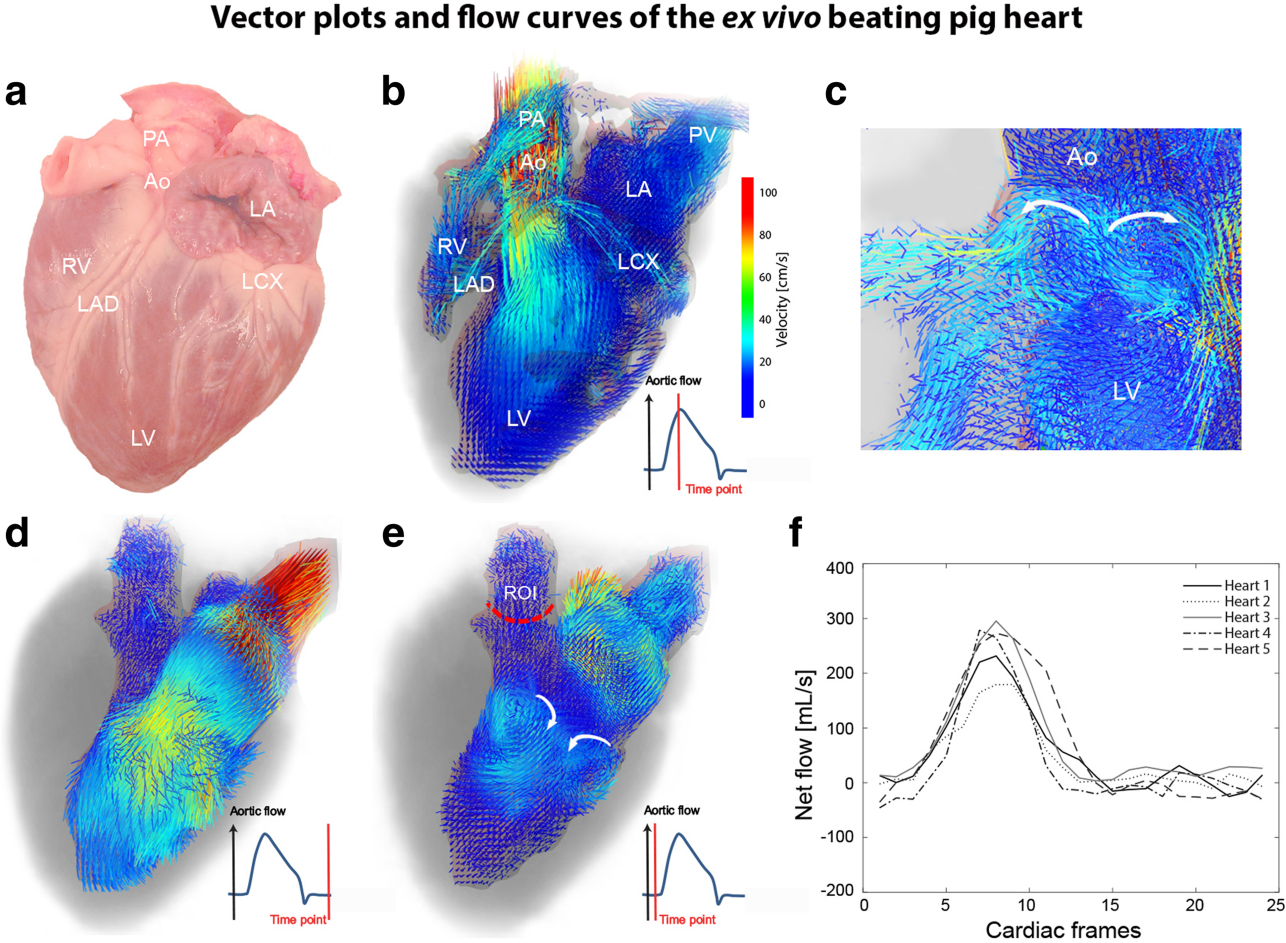
Velocity vector plots of one representative heart and aortic flow curves for all five hearts. **a** Anatomical reference of the heart before the experiment. **b** Vector plots of intracardiac flow in the ejection phase. **c** Vortex formation and coronary filling in the aortic root of the heart during diastole. For better visualisation of intracardiac flow, **d** shows the vector plots of the segmented LV at end-systole and **e** at end-diastole, where vortex formation can be observed. **f** Net flow downstream to the aortic valve for all five experiments, measured in an ROI indicated in **e**. *Ao* Aorta, *LA* Left atrium, *LAD* Left anterior descending artery, *LCX* Left circumflex artery, *LV* Left ventricle, *PA* Pulmonary artery, *PV* Pulmonary vein, *ROI* Region of interst, *RV* Right ventricle

For all seven hearts, the installation in the *ex vivo* beating pig heart platform and resuscitation was feasible. 4D flow MRI acquisitions were successful, and flow visualisation was feasible in all hearts. In Fig. 2, snapshot examples from one heart are shown. Figure 2a is a photograph of the heart before the MRI scan for anatomical reference. Figure 2b depicts reconstructed 4D flow MRI velocity vectors at peak systole. Velocity vectors of blood flow in the LV and the left atrium as well as coronary flow in the left anterior descending artery, the left circumflex artery, and the filling and ejection of the right ventricle were visualised. In the aortic root, late systolic vortex formation and filling of the coronary arteries were observed in all hearts as exemplified in Fig. 2c. Figure 2 d and e display velocity vectors of the LV at diastole and end-diastole in detail. In the left atrium and LV, filling and vortex formation after closing of the mitral valve were observed. Quantification of net flow *Q*(*t*) could be performed in ROIs downstream to the aortic valve (indicated in Fig. 2e), resulting in similar flow curves for all hearts as shown in Fig. 2f.

**Fig. 3:**
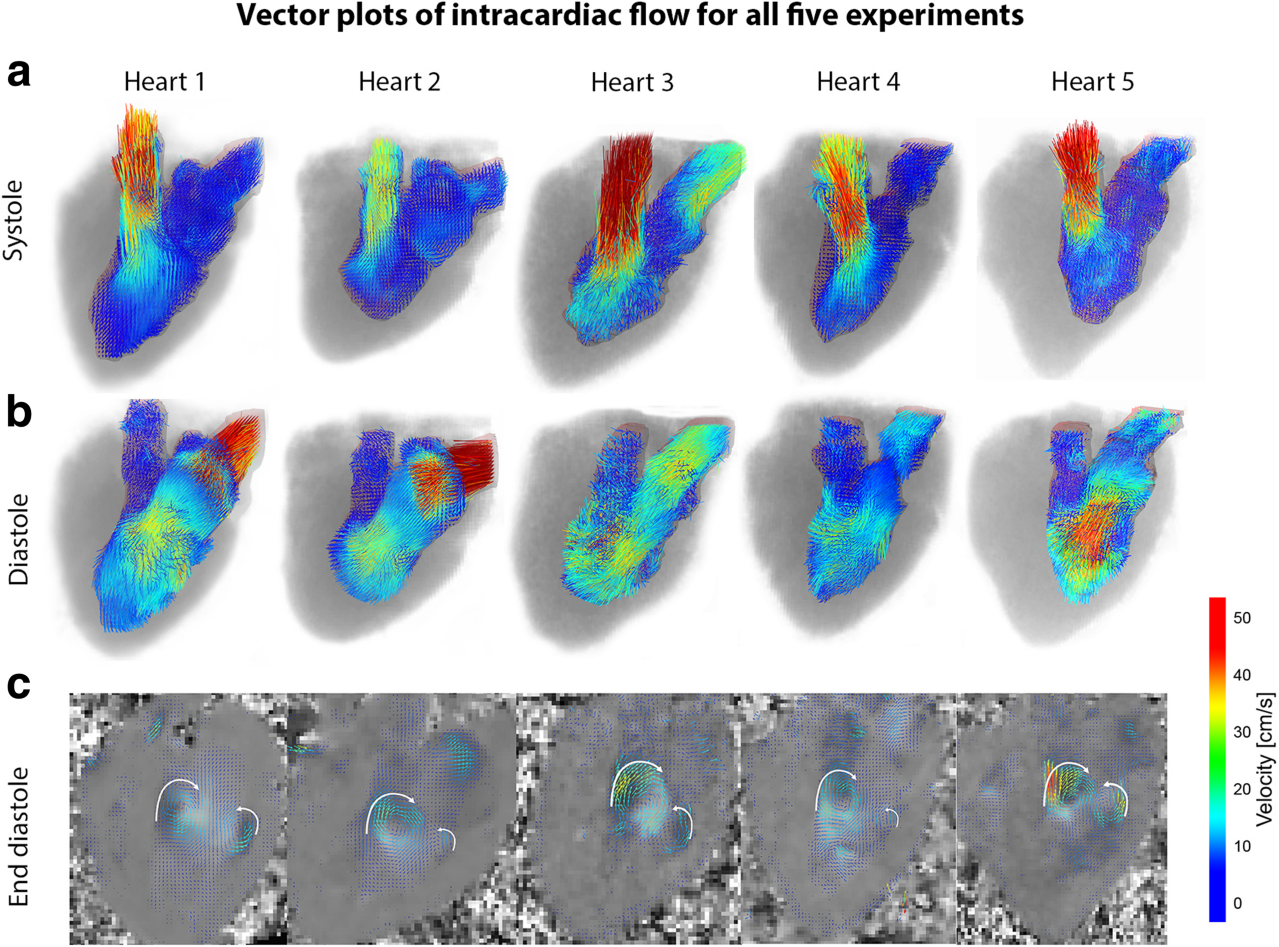
Velocity vector plots of all five experiments at three time points in the cardiac cycle. Vector plots for the segmented left ventricle (LV) of all five experiments during (**a**) systole and (**b**) diastole. During end-diastole (**c**), vortex formation could be observed in all five hearts

Velocity vectors in the LV of all five hearts are presented in Fig. 3, showing filling and ejection of blood in the LV. In all hearts, late diastolic vortex formation was found as shown in the vector plots in Fig. 3c. A video of the vector plots over the cardiac cycle can be found in Additional file 2: Video S2. In Table 1, HR, SV, CO, and regurgitation fraction for these hearts are given. The cardiac functional parameters, averaged for all five valves, were HR = 105 ± 12 bpm, SV_flow_ = 31 ± 6 mL, CO_flow_ = 3.3 ± 0.9 L/min, and regurgitation fraction = 16 ± 9%. Aortic annulus sizes were on average 31 ± 1 mm (mean ± standard deviation) for the long axis, 24 ± 2 mm for the short axis, and 600 ± 31 mm^2^ for the area.

**Table 1:** Cardiac parameters for the five hearts

		Heart 1	Heart 2	Heart 3	Heart 4	Heart 5	Mean ± standard deviation
Heart rate	(bpm)	104	98	109	93	123	105 ± 12
Stroke volume _flow_	(mL)	31	27	41	25	32	31 ± 6
Cardiac output _flow_	(L/min)	3.2	2.6	4.5	2.3	3.9	3.3 ± 0.9
Regurgitation fraction	(%)	11	19	5	30	16	16 ± 9
End-systolic volume	(mL)	101	122	59	31	71	77 ± 36
End-diastolic volume	(mL)	143	158	113	72	110	119 ± 33
Ejection fraction	(%)	29	22	48	57	36	38 ± 14
Stroke volume_volume_	(mL)	42	35	54	41	39	42 ± 7
Cardiac output_volume_	(L/min)	4.4	3.5	5.9	3.8	4.9	4.5 ± 1.0
Annulus size (long axis, short axis)	(mm)	31, 24	32, 23	31, 27	29, 22	31, 22	31 ± 1, 24 ± 2

Heart rate, stroke volume, and cardiac output were calculated from the aortic flow and from left ventricular volume segmentations. Regurgitation fraction was calculated from the aortic flow, and ejection fraction from the volumetric measurements. Aortic annulus diameters were measured during peak systole

**Fig. 4:**
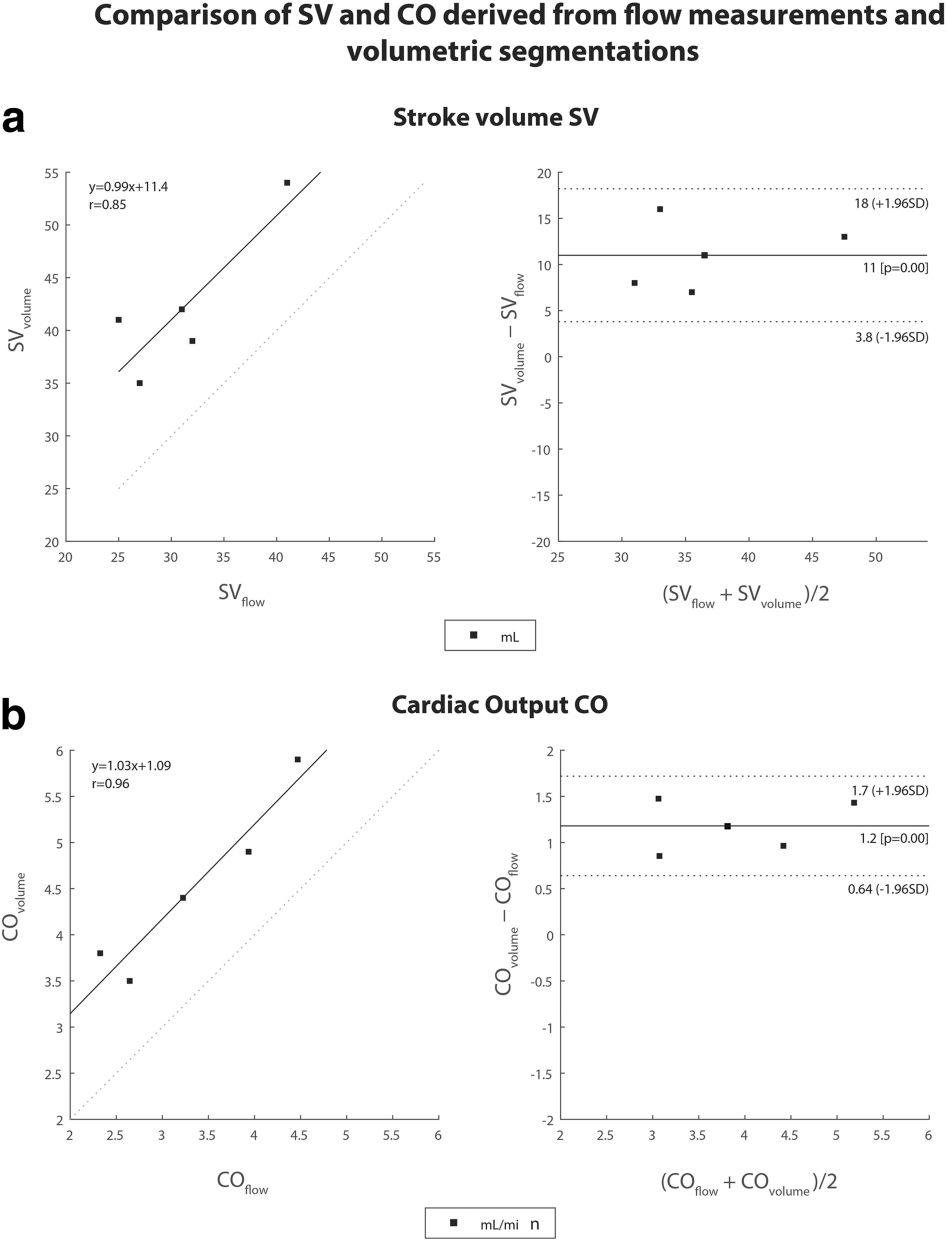
Linear regression and Bland-Altman plots for stroke volume and cardiac output calculated from flow and from volumetric measurements. Comparison of the two techniques used for cardiac output (CO) and stroke volume (SV) estimation using Bland-Altman analysis. **a** Linear regression and Bland-Altman plots for SV calculated from aortic flow data (SV_flow_) and from left ventricle (LV) volume (SV_volume_). **b** Linear regression and Bland-Altman plots for CO calculated from aortic flow data (CO_flow_) and from LV volume (CO_volume_)

Figure 4 shows Bland-Altman plots for CO and SV calculated from flow data and LV volume segmentations. The SV_volume_ calculated from LV volume was on average 11 mL higher than the SV_flow_ calculated from flow data. CO_volume_ was thus on average 1.2 L/min higher than CO_flow_.

The results of the flow curves, HR, CO_flow_ , and SV_flow_ for the one heart where the 4D flow MRI scan was repeated three times within the same scan session at 0 min, 12 min, and 1 h 5 min are summarised in Additional file 3: Figure S1. These parameters were similar for all three scans.

The results of the two hearts in which a TAVR procedure was performed are summarised in Additional files 4, 5, 6, 7 and 8.

## Discussion

In this study, 4D flow MRI was successfully applied in five pig hearts independently beating after installation and resuscitation in an MRI-compatible *ex vivo* beating pig heart platform. The platform provided detailed, comprehensive visualisations of time-resolved 3D, *i.e*., 4D, intracardiac blood flow and quantification of cardiac functional parameters. In literature, only two other setups have demonstrated *ex vivo* beating pig hearts in an MRI-compatible setup [3, 21], none of them using 4D flow MRI, as these scans can be very time consuming. In fact, advanced acceleration and reconstruction techniques are necessary to perform these scans in a feasible acquisition time. Additionally, the use of slaughterhouse byproducts as shown in this study is beneficial to reduce the number of *in vivo* animal experiments in view of ethical considerations as well as animal housing labour and costs.

The feasibility of the *ex vivo* beating pig heart platform for cardiac intervention was demonstrated in two additional experiments in which a TAVR procedure was conducted. In these TAVR experiments, 4D flow MRI was able to quantify and visualise the type and extent of aortic regurgitations, a typical complication after valve replacement procedure that is difficult to measure with echocardiography [15, 16].

Literature values for the SV of a healthy 60-kg pig’s LV are typically 65–81 mL [23] (in humans, 95 mL [24]). Literature values for the porcine HR are typically 85–114 bpm [23] (in humans, 60–100 bpm). This results in a porcine CO of 5–10 L/min [25] in comparison to a 4–8 L/min in humans. However, in our *ex vivo* beating pig heart platform, CO was artificially regulated via the preload, independently of the actual heart size. Therefore, at an experimental HR of 100–130 bpm, the porcine SV of the experiments shown in this study was expected to be lower and around 38–50 mL. Additionally, the SV calculated from flow measurements was slightly lower than that from volumetric measurements, since coronary flow could not be considered. In spite of the feasibility of visualising coronary flow, quantification was hampered by heart motion and spatial resolution.

In *in vivo* 4D flow MRI experiments of 12 juvenile pigs, Cesarovic et al. [17] found a stress HR of 124 ± 3 bpm which matched our experimental HR, similar also to the experimental HR reported by Vaillant et al. [21]. In their experiments, they found EDV = 55 ± 8 mL, ESV = 19 ± 6 mL, SV = 35 ± 4 mL, and an EF = 65% ± 7%. However, the difference between the *in vivo* experiment and the *ex vivo* beating pig heart experiment from this study is that pigs slaughtered for human consumption were used for the *ex vivo* model. The pigs reached a final live weight of approximately 110 kg, which is much more than a juvenile pig (30 kg). That weight difference was expressed in the large ESV and EDV measured in this experiment.

In general, complex flow patterns (such as vortices) are more difficult to capture with echocardiography as with 4D flow MRI. In our study, two late diastolic vortices were observed in the LV of all hearts (Figs. 2e and 3c). These vortices were also described by Elbaz et al. [8], who reported on vortex flow analysis during LV filling using 4D flow MRI in normal humans, and by Witschey et al. [18], who studied LV flow dynamics using 4D flow MRI in sheep.

This study has some limitations. The main idea was to use slaughterhouse waste material to allow an easy and cost-efficient setup. However, the use of pig hearts from large pigs raised for human consumptions limits the similarity between pig and human hearts. Additionally, an artificial human-like CO could limit the viability of the heart, which is why it might be an option to regulate the CO to another value. The hearts stayed vital for approximately 5 h, however would not survive several resuscitations, which limits their use for pre-procedure *versus* post-procedure measurements in the case of TAVR or any other intervention. As described by Driessen et al. [26], other cardiac MRI techniques such as black-blood, bright blood-balanced steady-state-free precession, perfusion, angiography, and T1-mapping sequences, are important to describe the full extent of cardiac function. As real blood is used, the *ex vivo* beating pig heart platform can be used for many of these MRI techniques, as well as for contrast-enhanced imaging, *e.g*., late gadolinium enhancement, as contrast agent could simply be added to the preload. A limitation for MRI techniques in general, however, could be the missing surrounding tissue, improving static phase offset corrections or shimming and which could also cause susceptibility artefacts. Also, the attachment of pacemaker lead cables on the heart’s surface can create some distortions. In general, the setup is also compatible with standard echocardiography instruments as well as pressure sensors and optical flow sensors. Another limitation is the use of five pig hearts in this feasibility study. The limited number of samples exaggerates the variation shown in the Bland-Altman plots. Including more hearts could reduce this variability.

An advantage of the isolated beating pig heart setup for MRI is that receiver radiofrequency coils can be placed close to the hearts’ surface, which results in high signal to noise and a high-flow contrast, even in very small anatomical structures such as the coronary arteries. The relatively small differences in velocity fields between the five hearts indicate good reproducibility of the experiments.

As shown in Additional files 4, 5, 6, 7 and 8 this platform has a potential for experiments on the performance of prosthetic valves or surgical strategies. Furthermore, with the isolated beating pig heart platform, aortic, pulmonary, and mitral regurgitation can be simulated. Complex settings of multiple regurgitations can be tested and investigated using 4D flow MRI. This is important since for example mitral regurgitation is common in patients with severe aortic regurgitation and aortic stenosis [27].

In conclusion, this study demonstrated the feasibility of 4D flow MRI data in a physiological working pig heart model pumping real blood and simulating physiological conditions. The isolated beating pig heart platform can allow for investigating new MRI sequences, pathophysiologic haemodynamics in relation with implanted artificial heart valves or also drug administration.

## Additional files

Additional file 1: **Video S1.** Isolated beating pig heart during Langendorff perfusion. Video of the resuscitated, beating pig heart installed in the experimental setup during Langendorff perfusion. (MP4 546 kb)

Additional file 2: **Video S2.** Velocity vectors in a representative heart throughout the cardiac cycle. Velocity vectors for one average cardiac cycle of an exemplary heart. The segmentation includes the left ventricle, the right ventricle, and the coronary arteries. Velocities range from 0 to 100 cm/s. (MP4 1590 kb)

Additional file 3: **Figure S1.** Repeated 4D flow MRI measurements of the same heart with a native valve. The heart was scanned three times, at time points 0 min, 12 min, and 1 h 5 min. The flow curves in the aorta are similar for all three scans, as well as HR, CO_flow, and SV_flow. (JPG 845 kb)

Additional file 4: Background, methods, and results of TAVR experiments conducted in two additional hearts. EURE-D-19-00017_ESM.docx. (DOCX 21 kb)

Additional file 5: **Figure S2.** Comparison of vector plots and flow curves in hearts with native and TAVR valves during systole and diastole. This figure shows velocity vectors visualised using GTFlow (Gyrotools, Zurich, Switzerland), comparing a heart with native and the hearts with implanted TAVR valves. For better understanding photographs of the valves were placed at the areas of signal loss. Flow curves were calculated per heart from mean velocities in an ROI drawn in the aorta, close to the aortic valve, showing a large backward flow for the Edwards valve. Vector plots of a heart with a native valve (left), the CoreValve (middle), and the Edwards valve (right) at (**a**) peak systole and (**b**) diastole. A high velocity regurgitation jet downstream to the Edwards valve as a consequence of paravalvular leakage can be seen during diastole. **c** Aortic (blue) net flow, (red) forward flow, and (grey) backward flow, showing no regurgitation for the native valve (left), moderate regurgitation for the CoreValve (middle), and severe regurgitation for the Edwards valve (right). (JPG 1420 kb)

Additional file 6: **Figure S3.** Visualisation of paravalvular leakage in the Edwards valve. Visualisation of aortic and mitral regurgitation jets in the heart with the Edwards valve using phase contrast data (feet-head encoding), vector plots and throughflow planes. **a** Short axis view of the heart with the CoreValve (left) and the Edwards valve (right) during diastole at the height of the left ventricular outflow tract. In phase contrast images PVL can be seen as high velocities (brighter areas) flowing back in the LV. **b** Edwards valve: Phase contrast data (two chamber view with feet-head flow encoding) and vector plots (**c**) for aortic regurgitation during diastole (top) and mitral regurgitation (bottom) during systole. **d** Edwards valve: multiple eccentric regurgitation jets at the position of the prosthetic aortic valve and one central regurgitation jet in the mitral valve. (JPG 1400 kb)

Additional file 7: **Video S1.** Velocity vectors in the heart with CoreValve throughout the cardiac cycle. Velocity vectors for one average cardiac cycle of the CoreValve. The segmentation includes the LV and velocities range from 0–100 cm/s. In the region of the valve no velocity vectors can be seen due to susceptibility artifacts. (MP4 1670 kb)

Additional file 8: **Video S2.** Velocity vectors in the heart with Edwards valve throughout the cardiac cycle. Velocity vectors for one average cardiac cycle of the Edwards valve. The segmentation includes the LV and velocities range from 0–100 cm/s. In the region of the valve no velocity vectors can be seen due to susceptibility artifacts, however paravalvular leakage is visible as a regurgitation jet during diastole. (MP4 1580 kb)

## Data Availability

The datasets used and/or analysed during the current study are available from the corresponding author on reasonable request.

## Abbreviations

3D: Three dimensional
4D: Four dimensional
bpm: Beats per minute
CO: Cardiac output
EDV: End-diastolic volume
EF: Ejection fraction
ESV: End-systolic volume
HR: Heart rate
LV: Left ventricle
MRI: Magnetic resonance imaging
ROI: Region of interest
SV: Stroke volume
TAVR: Transcatheter aortic valve replacement
