# Supplementary figures and images for: An isolated beating pig heart platform for a comprehensive evaluation of intracardiac blood flow with 4D flow MRI: a feasibility study
Source: Eur Radiol Exp. 2019 Oct 25;3:40. doi: 10.1186/s41747-019-0114-5 (PMC6813403; doi:10.1186/s41747-019-0114-5)

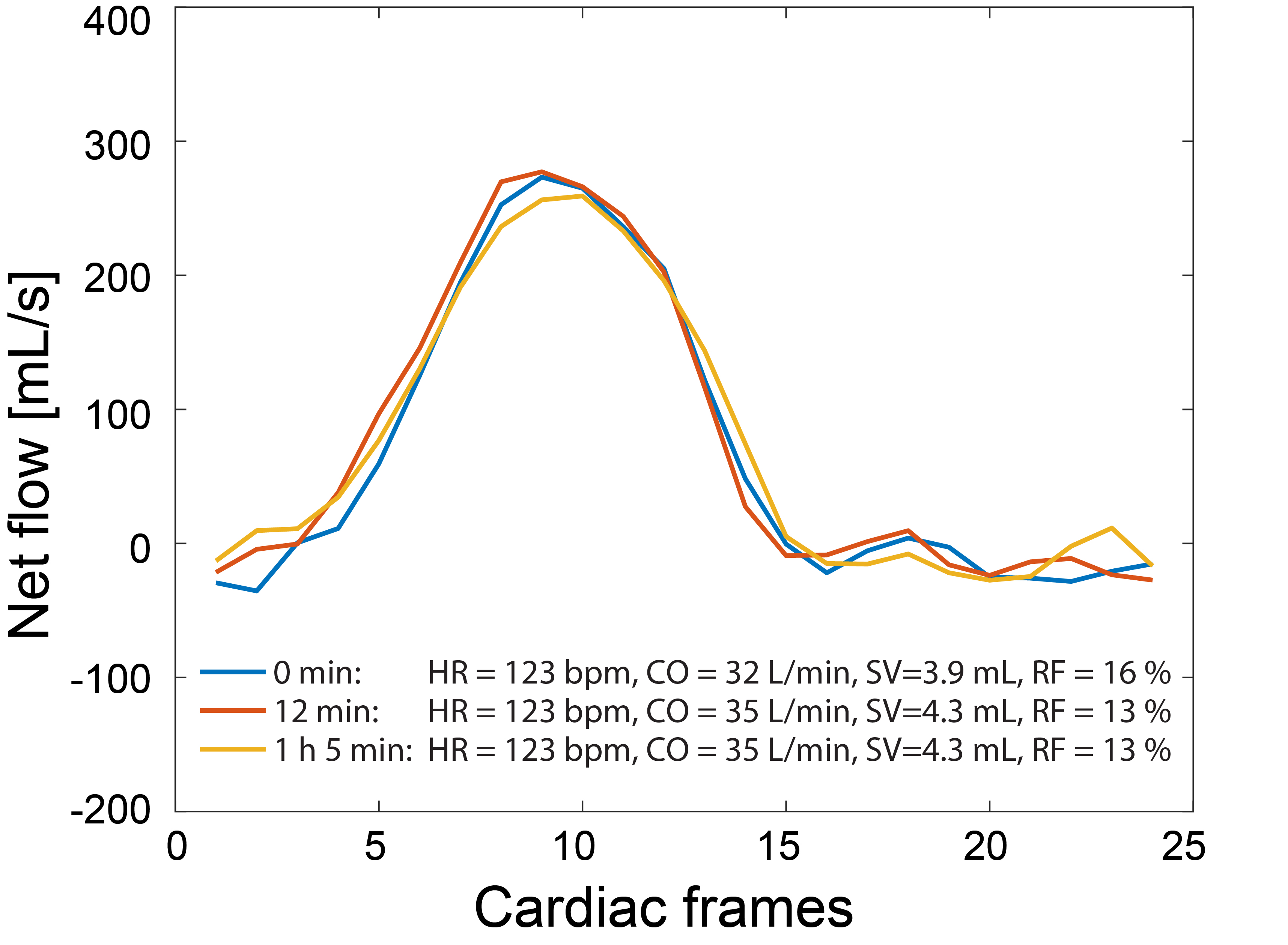

Supplement: Supplementary file 3 — Figure S1. Repeated 4D flow MRI measurements of the same heart with a native valve. The heart was scanned three times, at time points 0 min, 12 min, and 1 h 5 min. The flow curves in the aorta are similar for all three scans, as well as HR, CO_flow, and SV_flow. (JPG 845 kb) [file 41747_2019_114_MOESM3_ESM.jpg]

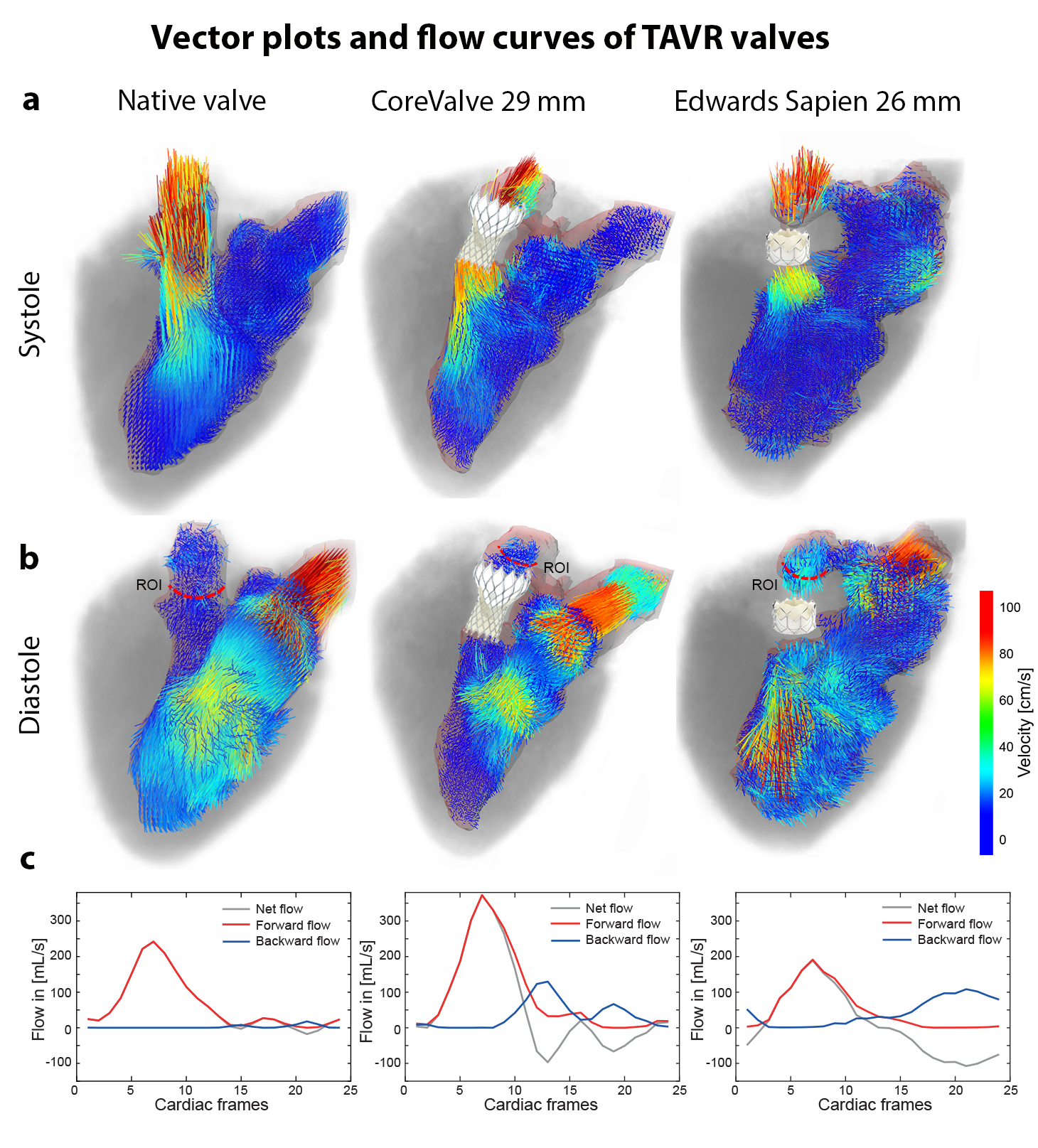

Supplement: Supplementary file 5 — Figure S2. Comparison of vector plots and flow curves in hearts with native and TAVR valves during systole and diastole. This figure shows velocity vectors visualised using GTFlow (Gyrotools, Zurich, Switzerland), comparing a heart with native and the hearts with implanted TAVR valves. For better understanding photographs of the valves were placed at the areas of signal loss. Flow curves were calculated per heart from mean velocities in an ROI drawn in the aorta, close to the aortic valve, showing a large backward flow for the Edwards valve. Vector plots of a heart with a native valve (left), the CoreValve (middle), and the Edwards valve (right) at (a) peak systole and (b) diastole. A high velocity regurgitation jet downstream to the Edwards valve as a consequence of paravalvular leakage can be seen during diastole. c Aortic (blue) net flow, (red) forward flow, and (grey) backward flow, showing no regurgitation for the native valve (left), moderate regurgitation for the CoreValve (middle), and severe regurgitation for the Edwards valve (right). (JPG 1420 kb) [file 41747_2019_114_MOESM5_ESM.jpg]

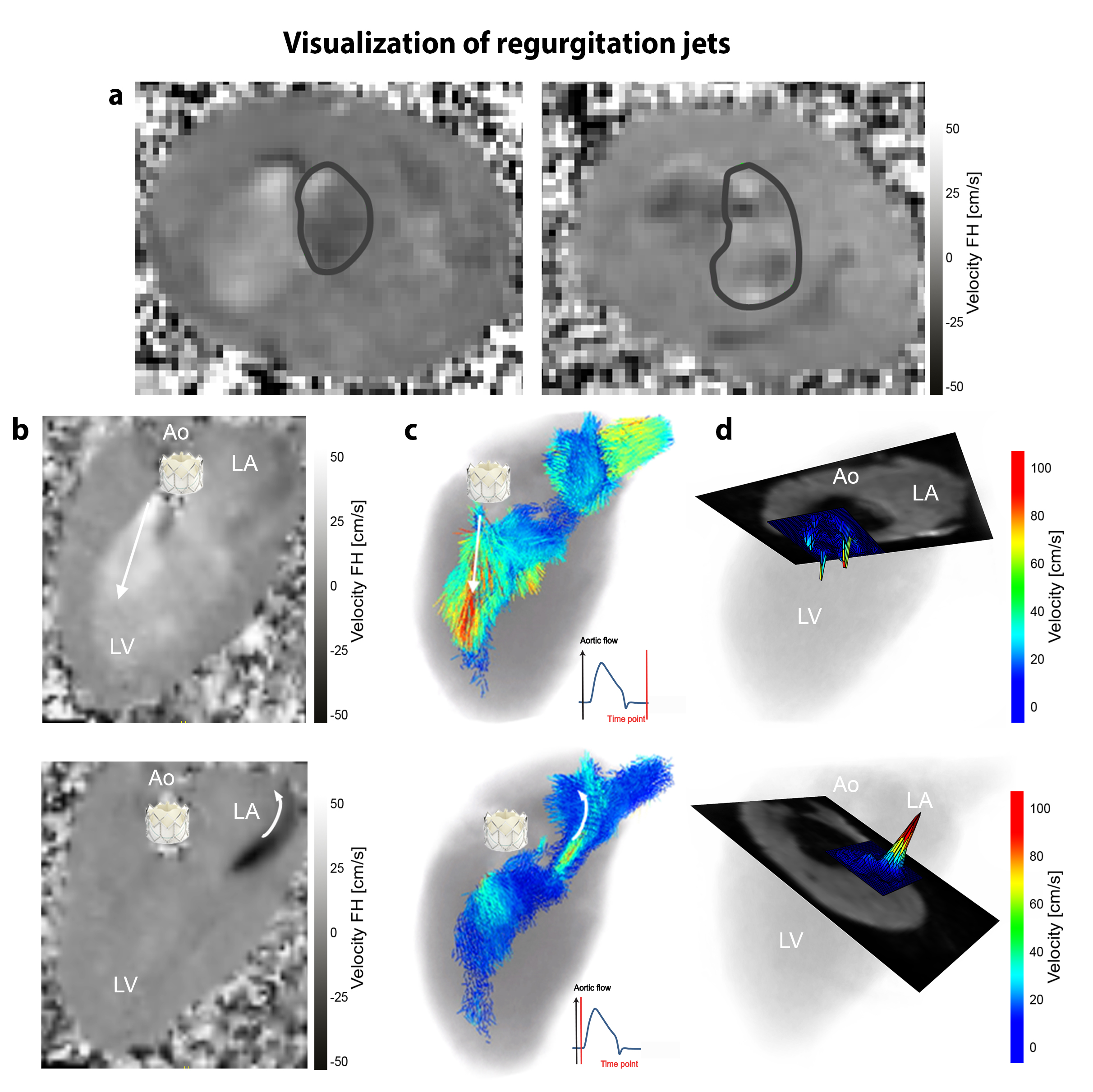

Supplement: Supplementary file 6 — Figure S3. Visualisation of paravalvular leakage in the Edwards valve. Visualisation of aortic and mitral regurgitation jets in the heart with the Edwards valve using phase contrast data (feet-head encoding), vector plots and throughflow planes. a Short axis view of the heart with the CoreValve (left) and the Edwards valve (right) during diastole at the height of the left ventricular outflow tract. In phase contrast images PVL can be seen as high velocities (brighter areas) flowing back in the LV. b Edwards valve: Phase contrast data (two chamber view with feet-head flow encoding) and vector plots (c) for aortic regurgitation during diastole (top) and mitral regurgitation (bottom) during systole. d Edwards valve: multiple eccentric regurgitation jets at the position of the prosthetic aortic valve and one central regurgitation jet in the mitral valve. (JPG 1400 kb) [file 41747_2019_114_MOESM6_ESM.jpg]
