# Supplementary material for: An isolated beating pig heart platform for a comprehensive evaluation of intracardiac blood flow with 4D flow MRI: a feasibility study
Source: Eur Radiol Exp. 2019 Oct 25;3:40. doi: 10.1186/s41747-019-0114-5 (PMC6813403; doi:10.1186/s41747-019-0114-5)
Supplement: Supplementary file 4 — Background, methods, and results of TAVR experiments conducted in two additional hearts. EURE-D-19-00017_ESM.docx. (DOCX 21 kb) [file 41747_2019_114_MOESM4_ESM.docx]

**ADDITIONAL MATERIAL**

Transcatheter aortic valve replacement (TAVR) is a valve replacement procedure in which a biological prosthetic valve is delivered by a catheter and implanted in the failing native aortic valve. For high-risk patients with symptomatic aortic stenosis (AS), TAVR is the therapy of choice leading to better survival rates when compared to open heart surgery [1, 2]. Although generally beneficial, valve replacements are not without complications. Paravalvular leakage (PVL) or less frequent regurgitations of blood through the center of the valve may occur (prosthetic valve regurgitation) [3].

In addition to the five hearts with native valves, we investigated the feasibility of TAVR in the isolated beating pig heart platform. For this purpose, we replaced the native valves in two additional experiments by TAVR valves. Although TAVR procedure is strenuous for the organ, both hearts could be resuscitated after being installed in the isolated beating pig heart platform and 4D flow MRI could be performed successfully.

Cardiac parameters for CoreValve and Edwards valve were $\mathrm{SV}_{\mathrm{CoreValve}}$=27 mL, $\mathrm{CO}_{\mathrm{CoreValve}}$=3.5 L/min, RF_CoreValve_=37% and $\mathrm{SV}_{\mathrm{Edwards}}$=4 mL, $\mathrm{CO}_{\mathrm{Edwards}}$= 0.5 L/min, RF_Edwards_=86%, respectively. Aortic regurgitation (AR) grading [1, 4] indicated a moderate AR (30% < RF_CoreValve_=37% < 60%) for the CoreValve and a severe AR for the Edwards valve (RF_Edwards_=86% > 60%). Similar to the native hearts, velocity vectors could be visualized in both hearts with prosthetic valves. Due to susceptibility mismatch and shielding, there was signal loss at the site of the prosthetic valves. In Additional file 5: Supplemental figure 2a-b, velocity vectors at peak systole and end diastole are shown for an exemplary native heart and the hearts with both prosthetic valves. Velocity vector plots (Additional file 5: Supplemental figure 2b, right) indicated a small regurgitation jet for the CoreValve and a large regurgitation jet for the Edwards valve during diastole with local velocities up to 180 cm/s. Additional file 5: Supplemental figure 2c summarizes net-, forward-, and backward flow through the valves during the cardiac cycle (measured in ROIs indicated in Additional file 5: Supplemental figure 2b), showing high backflow for the Edwards valve. Video files containing vector plots of the cardiac cycle for the CoreValve and the Edwards valve can be found in Additional file 7: Supplemental video 1 and Additional file 8: Supplemental video 2.

Both TAVR valves did not show regurgitation due to transvalvular regurgitation, but due to PVL, which is characterized by regurgitating blood between cardiac tissue and the prosthetic valve. PVL was visible as (multiple) regurgitation jets at the border between valve and cardiac tissue, e.g. as shown for the Edwards valve in Additional file 6: Supplemental figure 3. Furthermore, mitral regurgitation (MR) was found in the heart with the Edwards valve.

In the two additional TAVR experiments, for both implanted TAVR valves PVL was observed, likely created by insufficient sealing in the absence of aortic valve calcification [5] or due to small prosthetic valve sizes (29 mm and 26 mm) and migration of the valve [3]. In general, the diameter of the aortic annulus of pig and human hearts are comparable (25-30 mm) [6, 7], which allows for the use of human TAVR implants in pig hearts. The clinical indication for TAVR procedure is a calcified AS to achieve a stable position inside the native valve, since an excessive radial force is needed to create sufficient sealing [5]. The prosthetic valves available were not larger than the aortic annulus as in common clinical practice in order to create sufficient sealing. PVL was further exacerbated by the absence of a calcified annulus or valve [5, 8]. Additionally, regurgitations observed over the mitral valve were most likely created by the altered loading conditions of the LV with a higher volume and pressure load. Even with insufficient sealing of the TAVR valves still the feasibility of TAVR in the isolated peating pig heart model could be shown. Moreover, the advantages of 4D flow MRI in typical complications after TAVR procedure could be demonstrated, by providing quantitative and visual descriptions of the regurgitation jets.

**References**

1. Nishimura RA, Otto CM, Bonow RO, et al (2014) 2014 AHA/ACC guideline for the management of patients with valvular heart disease: executive summary: a report of the american college of cardiology/american heart association task force on practice guidelines. Circulation 129: . doi: 10.1161/CIR.0000000000000029/-/DC1

2. Rahimtoola SH (2010) Choice of prosthetic heart valve in adults. J Am Coll Cardiol 55: . doi: 10.1016/j.jacc.2009.10.085

3. Smolka G, Wojakowski W (2010) Paravalvular leak - important complication after implantation of prosthetic valve. E-Journal Cardiol Pract 9:1–7

4. Maurer G (2006) Aortic regurgitation. Heart 92:994–1000 . doi: 10.1136/hrt.2004.042614

5. Haensig M, Rastan AJ (2012) Aortic valve calcium load before TAVI: is it important? Ann Cardiothorac Surg 1:160–164 . doi: 10.3978/j.issn.2225-319X.2012.06.02

6. Sands MP, Rittenhouse EA, Mohri H, Merendino KA (1969) An anatomical comparison of human, pig, calf, and sheep aortic valves. Ann Thorac Surg 8: . doi: doi.org/10.1016/S0003-4975(10)66071-7

7. Iaizzo PA Handbook of Cardiac Anatomy, Physiology, and Devices. Springer

8. de Weger A, van Tuijl S, Stijnen M, et al (2010) Images in cardiovascular medicine direct endoscopic visual assessment of a transcatheter aortic valve implantation and performance in the PhysioHeart, an isolated working heart platform. Images Cardiovasc Med 121:261–263 . doi: 10.1161/CIR.0b013e3181d9b879
